# Cognitive function among women with breast cancer receiving endocrine therapy: what are the impacts?

**DOI:** 10.1093/jncics/pkad026

**Published:** 2023-03-25

**Authors:** Joanna E Fardell, Adam Walker, Raymond J Chan, Janette L Vardy

**Affiliations:** University of New South Wales Medicine and Health, UNSW Sydney, Australia; Western Sydney Youth Cancer Service, Westmead Hospital, Sydney, Australia; Laboratory of Immuno Psychiatry, Neuroscience Research Australia, Randwick, Australia; Discipline of Psychiatry and Mental Health, University of New South Wales, Sydney, Australia; Caring Futures Institute, Flinders University, Adelaide, Australia; Faculty of Medicine and Health, University of Sydney, Sydney, Australia; Concord Cancer Centre, Concord Repatriation General Hospital, Sydney, Australia

Endocrine therapy (ET) is well established as a treatment in hormone-sensitive breast cancers, which account for approximately 75% of breast cancers. Commonly used endocrine treatments include selective estrogen receptor (ER) modulators (eg, tamoxifen), selective ER degraders (eg, fulvestrant), aromatase inhibitors (AIs; eg, letrozole, anastrozole, exemestane), and ovarian function suppression (eg, goserelin). There is strong evidence to support AIs as the preferred adjuvant treatment for postmenopausal women with ER–positive (ER+) breast cancer, with reductions in breast cancer recurrence and mortality compared with women receiving tamoxifen (1). Options for premenopausal women are tamoxifen or an AI with ovarian suppression (2).

Despite the benefits of ET on disease recurrence and mortality, many women discontinue treatment early (3). Reasons for nonadherence are complex, but side effects, particularly arthralgias with AIs (4) and hot flushes with tamoxifen, are common reasons given for discontinuation (5). Cognitive symptoms are also reported. Most of the research into cancer-related cognitive impairment (CRCI) has been in women with breast cancer who have had chemotherapy before commencing ET. The extant literature suggests cognitive symptoms are more common with chemotherapy than ET. A survey of 2296 women (aged 34-82 years) with breast cancer found that 60% reported cognitive symptoms after receiving treatment (6). Cognitive symptoms were greater in women who received chemotherapy only compared with hormone treatment only (adjusted odds ratio = 5.63, 95% confidence interval = 3.52 to 9.00 vs 1.64, 95% confidence interval = 1.15 to 2.33). Rates of cognitive impairment based on formal neuropsychological tests in women on ET vary from 32% to 64% (7,8).

We commend Kjoe and colleagues (9) on their important study evaluating the longer-term effects of tamoxifen followed by exemestane or exemestane alone on cognitive function in the Tamoxifen and Exemestane Adjuvant Multinational trial. Participants were women with postmenopausal hormone receptor–positive breast cancer. They were randomized to either sequential treatment with tamoxifen for 2.5-3 years followed by exemestane or exemestane for 5 years. A side study assessed cognitive function in 206 breast cancer patients who were chemotherapy-naïve before commencing ET and in 124 age-matched noncancer control participants at baseline, 1 year (previously reported) (10), and 5 years, and included formal neuropsychological tests and cognitive symptoms. After controlling for age, IQ, attrition, menopausal symptoms, anxiety or depression, and/or fatigue, they found worse outcomes in the sequential group (tamoxifen to exemestane) compared with controls on verbal memory and executive function at 1 and 5 years, with more decline at 1 year on information processing speed and executive function, and long-term decline on verbal memory compared with the exemestane group. There was no cognitive decline in the exemestane group compared with controls. As stated by the authors, their results suggest a carry-over effect of tamoxifen on cognitive function even after switching to exemestane. It should be noted that the effect sizes were small (<0.38) but likely to be noticeable to survivors, particularly those with higher cognitive demands.

The results presented by Kjoe et al. (9) are largely consistent with the extant literature. In a review of impacts on cognitive domains affected by ET for people with breast cancer, global measures of cognitive function are rarely implicated. However, memory, and in particular verbal memory, and executive function as measured by performance on measures of verbal fluency have consistently shown deficits as a result of ET (11). Equivocal evidence exists for impact on processing speed, whereas other domains of cognition appear unaffected (11). Kjoe et al. (9) found short-term decrements in information processing speed, but not reaction speed or motor speed, in the sequential group only. Similarly, the sequential group evidenced reduced verbal memory as indicated by mean performance across short- and long-delay list learning and a visual–verbal paired association task. In contrast, verbal fluency was largely unchanged across time, with no difference compared with controls or between sequential or exemestane groups. However, executive function, as measured by mean performance across Trail Making Test B and Stroop card 3, was found to be impaired in the sequential group. These executive function tests both involve category switching, inhibition, and speeded responding. The role of reduced processing speed and fatigue (mental and physical) may be important for understanding these results, and although the authors report a diminishing self-reported fatigue over time, inspection of mean scores as presented in Figure 2 indicates fatigue continues to be an issue for some participants at 5 years of ET therapy. In addition, further research exploring whether cognitive impairment is cumulative is important, given that the duration of ET for many women has increased; 5-10 years of treatment is recommended (12).

Baseline neuropsychological assessment indicated worse performance (ie, greater cognitive impairment) on some cognitive domains (10). Although not statistically significant, there was a trend for reduced cognitive function at baseline to further worsen at short-term (1 year) and long-term (5 years) follow-up for this sample of postmenopausal women with breast cancer compared with controls. This worsening of cognitive function appeared to occur despite opportunity for practice effects, and the presented results may underestimate impacts on cognitive function over time. However, in the absence of larger, sufficiently powered trials, this interpretation is speculative and warrants further validation.

Like previous cross-sectional studies among women receiving ET, cognitive symptoms were self-reported at baseline and short-term follow-up, before ET, and 1 year into ET (10,13). However, cognitive symptoms appeared to improve by 5 years of ET, particularly for the sequential group, suggesting a diminishing impact at a functional level. The European Organisation for Research and Treatment of Cancer Core Quality of Life Questionnaire (EORTC-QLQ)-C30 2-item cognitive function subscale was used to assess self-reported cognitive function. We have previously demonstrated scores below 75 are consistent with meaningful cognitive symptoms in a sample of 294 people with mixed cancer diagnoses but demonstrated inferior specificity compared with other cognitive function patient-reported outcome measures (14). As Kjoe et al. (9) acknowledge, further research using more comprehensive assessment of self-reported cognitive function and functional real-world outcomes is needed.

The study by Kjoe et al. (9) is important in providing 5-year longitudinal data of chemotherapy-naive breast cancer patients randomized in the main Tamoxifen and Exemestane Adjuvant Multinational study to sequential treatment (tamoxifen then exemestane) or exemestane. It needs to be noted that the cognitive study was in a smaller optional subgroup, with some baseline imbalances. Another limitation acknowledged by the study authors was that the 5-year cognitive assessment was not part of the original trial, and there was a 66% attrition between 12 months and 5 years for the sequential group and 43% for the exemestane group (9). As is common in cognitive studies, attrition was higher in patients likely to have lower cognitive reserves (lower IQ and older age). The potential effect of attrition bias is unknown. Although future larger studies were recommended by the study authors, it will be extremely challenging to conduct a similar randomized study in the postmenopausal setting given considerable benefits in the use of AIs in reducing recurrence and mortality, compared with tamoxifen (1), but comparison between different AI could be beneficial. Comprehensive assessments of CRCI require collection of extensive patient-reported outcomes and objective neuropsychological data, which can be burdensome for participants, especially if CRCI is studied as a secondary objective. Given the constant evolution of treatments, a more pragmatic design involving real-world ecological measures on a large cohort of breast cancer patients may be desirable.

Further research is also required to unpack the precise neurobiological mechanism(s) of cognitive impairment for tamoxifen vs exemestane in the hope that the next generation of anticancer ETs ensures cognitive function is preserved. Both tamoxifen and exemestane readily cross the blood-brain barrier but inhibit ER signalling via different mechanisms of action. In the brain, tamoxifen can act as an ER agonist or antagonist influenced by whether brain estradiol is depleted or abundant, and the impact of tamoxifen on cognition may be influenced by the individual’s ratio of brain ER subtypes α and β (15). Tamoxifen also appears to have pharmacological properties that may be independent of its interactions with ERs, including affecting dopamine transport and inhibition of protein kinase C, which are important for neurotransmission and neuroplasticity among other proposed non-ER–mediated mechanisms (16).

On the other hand, exemestane blocks the conversion of testosterone to estradiol. AIs can also exert brain region–specific effects and affect cognition (17,18) but perhaps to a lesser degree than ER antagonist or agonists like tamoxifen by avoiding some of tamoxifen’s off-target effects. Interestingly, the type of AI used in this study may have been fortuitous for patients. A preclinical study comparing exemestane with nonsteroidal AIs found that female mice treated with anastrozole and letrozole showed hippocampal-dependent memory impairments, whereas cognitive function in mice treated with exemestane was preserved (19), possibly due to the differences in the permanency of androgen conversion between exemestane and reversible nonsteroidal AIs as well as their proposed differential effects on cellular Wnt signalling (19). Finally, it is possible that the increased levels of brain testosterone that result from aromatase inhibition are beneficial for cognition given that testosterone supplementation has been investigated to improve cognitive aging, albeit mostly in men (20). Notably, AIs and especially tamoxifen have been implicated in many neural processes other than those listed here, and it is likely that the negative effects of tamoxifen on cognition are the result of many of these working in tandem.

Reductions in breast cancer recurrence and mortality support AIs over tamoxifen as the preferred adjuvant treatment for postmenopausal women with ER+ breast cancer (1). The findings of increased CRCI in postmenopausal women receiving tamoxifen compared with exemestane provide further support for using an AI where possible. However, tamoxifen is still commonly used in premenopausal women, in postmenopausal women who are intolerant of AIs, and in lower-income countries where AIs are not as readily available. Further research is required to determine the impact of different hormonal treatments on cognitive function in breast cancer survivors in both the premenopausal and postmenopausal setting.

## Data Availability

No new data were generated or analyzed for this editorial.
